# Evaluation of a seven gene mutational profile as a prognostic factor in a population-based study of clear cell renal cell carcinoma

**DOI:** 10.1038/s41598-022-10455-x

**Published:** 2022-04-20

**Authors:** Jeroen A. A. van de Pol, Paranita Ferronika, Helga Westers, Manon van Engeland, Martijn M. Terpstra, Kim M. Smits, Kim de Lange, Piet A. van den Brandt, Rolf H. Sijmons, Leo J. Schouten, Klaas Kok

**Affiliations:** 1 Department of Epidemiology, GROW-School for Oncology and Developmental Biology, Maastricht University, Peter Debyeplein 1, 6229 HA Maastricht, The Netherlands; 2 Department of Genetics, University of Groningen, University Medical Centre Groningen, Groningen, The Netherlands; 3 Department of Anatomical Pathology, Universitas Gadjah Mada, Faculty of Medicine, Public Health, and Nursing, Yogyakarta, Indonesia; 4 Department of Pathology, GROW-School for Oncology and Developmental Biology, Maastricht University Medical Centre, Maastricht, The Netherlands; 5 Department of Epidemiology, Care and Public Health Research Institute (CAPHRI), Maastricht University, Maastricht, The Netherlands

**Keywords:** Cancer epidemiology, Cancer genetics, Cancer, Renal cancer, Renal cell carcinoma

## Abstract

In this study, we investigate the influence of the seven genes (*VHL, PBRM1, SETD2, BAP1, KDM5C, MTOR* and *TP53*) most frequently mutated in clear cell renal cell cancer (ccRCC) on cancer-specific survival (CSS) in the prospective Netherlands Cohort Study on diet and cancer. DNA isolated from routinely archived formalin-fixed paraffin-embedded tumour blocks from 252 incident ccRCC cases was available for targeted next generation sequencing. Based on the sequencing quality and the completeness of information on clinical characteristics and follow-up, we could use 110 cases for survival analysis. The association with CSS for each mutated gene in these cases was tested using multivariable Cox proportional hazards models to estimate hazards ratios (HR) and confidence intervals (CIs), and we observed mutations in one or more of the seven genes in 64 out of 110 cases (58%). In the multivariable-adjusted analyses, mutations in *VHL* and *PBRM1* were associated with better CSS (HRs (95% CI) 0.34 (0.13‒0.89) and 0.17 (0.04–0.66), respectively), although these results were not statistically significant after multiple testing correction. No association was observed for the other five genes, which may be attributable to limited power.

## Introduction

Kidney cancer is an often lethal disease with a mortality rate of 3 per 100,000 population in Western Europe^1^. Renal cell cancer (RCC) accounts for approximately 90% of all kidney cancer, with clear cell renal cell carcinoma (ccRCC) being the most common RCC subtype (70%)^2,3^. Various clinical characteristics have been established as prognostic factors for ccRCC, including tumour size, stage, grade and necrosis^4^. In addition to these clinical characteristics, the mutational profile of the tumour may also be a prognostic factor. High-throughput sequencing databases such as the Catalogue of Somatic Mutations in Cancer (COSMIC) and The Cancer Genome Atlas (TCGA) project provide a clear overview of the genes that are frequently mutated in ccRCC^5,6^. Based on 512 ccRCC cases in the PanCancer study of TCGA, the most frequently mutated genes in ccRCC are: *VHL* (41%), *PBRM1* (38%), *SETD2* (12%), *BAP1* (10%), *MTOR* (8%) and *KDM5C* (5%)^6–8^. In addition, while the mutation rate of *TP53* in the TCGA is low (3%), the COSMIC database indicates that *TP53* may also be frequently mutated in ccRCC (7%)^9^.

These genes have been implicated in a number of ccRCC-related processes. Inactivation of the *VHL* tumour-suppressor gene leads to disrupted molecular oxygen sensing^10^, whereas *PBRM1, SETD2, KDM5C and BAP1* are chromatin-modifying proteins, and their inactivation is a prominent feature in the development of ccRCC^10–13^. *MTOR*, which is part of the PI3K/AKT/mTOR signalling pathway, is involved in multiple biological functions that regulate cell growth (e.g. cell survival, metabolism and proliferation) and represents a promising therapeutic target^14–16^. However, while the roles of all these genes in the development of ccRCC has been extensively investigated, there is less evidence regarding their role in ccRCC prognosis. In previous studies, mutations in *BAP1, SETD2*, *KDM5C* and *TP53* have been associated with an unfavourable prognosis in ccRCC^17–19^, but the *BAP1* association is the only one that has been consistently reported by several studies (Supplementary Table S1.1). No consistent associations with cancer-specific survival (CSS) have been found for *VHL, PBRM1* and *MTOR*^18–22^.

In the present study, we assessed the association between mutations in the most frequently mutated genes in ccRCC—*VHL*, *PBRM1*, *SETD2, BAP1, MTOR*, *KDM5C* and *TP53*—and CSS. In addition, we evaluated the association between the co-occurrence of mutations in these genes and CSS using data from the Netherlands Cohort Study on diet and cancer (NLCS), a large-scale population-based prospective cohort study with a long follow-up (23.3 years)^23^. A characteristic feature of the NLCS cohort is its treatment-naive population. Because surgery was the default ccRCC therapy and targeted therapies were not widely available in the period when most included patients were diagnosed (1986‒2008), only a few patients received adjuvant therapy in the first 3 months post-diagnosis.

## Results

### Targeted sequencing

**Figure 1:**
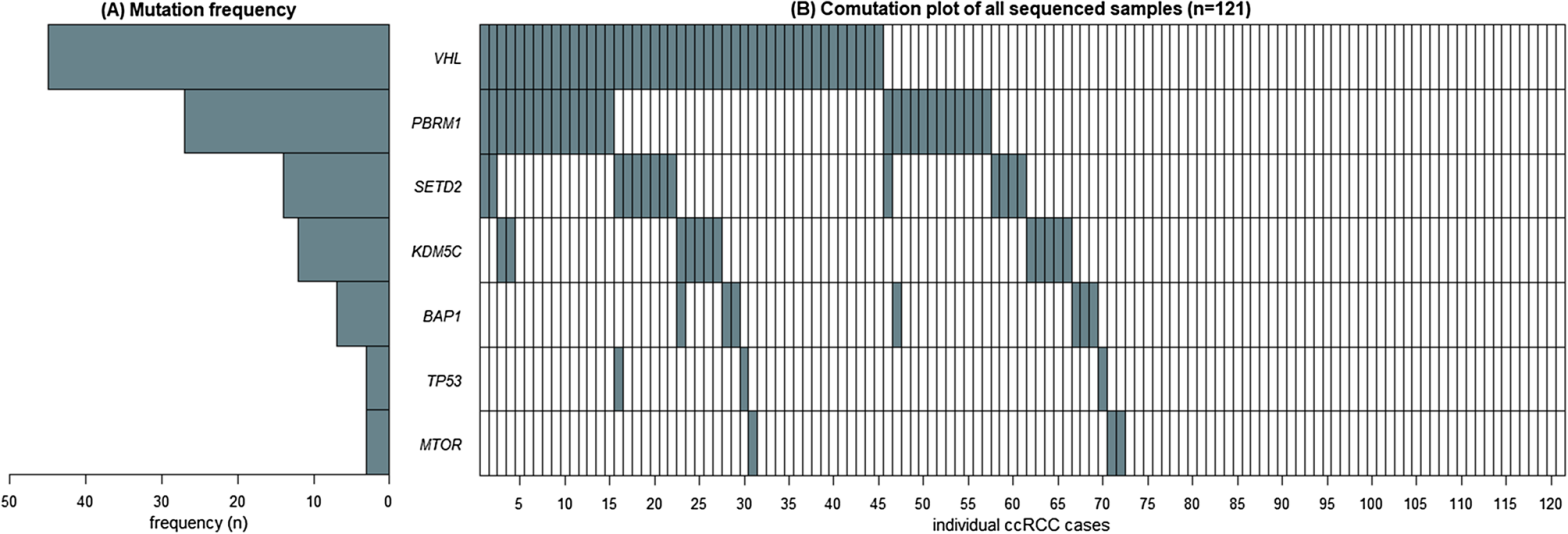
Combinations of mutations present in ccRCC cases in the Netherlands Cohort Study on diet and cancer (N = 121). (**A**) Mutation frequency for the seven selected genes. (**B**) Co-mutation plot of all sequenced samples. Each column represents one ccRCC case. Mutations in the individual genes labelled on the left are shown by the grey-blue bars.

The characteristics of the cases and samples and the mutation frequency of the genes included in the analysis are described in Table 1. Supplementary Table S2.1 provides an overview of sample and sequencing characteristics for all 252 sequenced samples and the 121 samples ultimately included in analyses. The mean duration of storage of the formalin-fixed paraffin-embedded (FFPE) tissue blocks was 9.8 years (SD 3.3), and a longer duration of storage before DNA isolation was associated with a lower DNA concentration in stock after DNA isolation, while the amount of DNA fragmentation was associated with a reduction in average read depth (Supplementary Table S2.2). Overall, 72 of the 121 (59.5%) cases had mutations in at least one of the seven target genes, and we identified 45 (37.2%) mutations in *VHL*, 27 (22.3%) in *PBRM1*, 14 (11.6%) in *SETD2*, 12 (9.9%) in *KDM5C*, 7 (5.8%) in *BAP1* was, 3 (2.5%) in *MTOR* and 3 (2.5%) in *TP53* (see Fig. 1).

**Table 1:** Characteristics of ccRCC cases in the Netherlands Cohort Study on diet and cancer (1986–2008).

Characteristics	Cohort, n = 121	
Age at diagnosis, years, median (range)	71	(57–88)
Year of cancer diagnosis, median (range)	1996	(1986–2008)
Survival, months, median (range)	68	(0–245)
**Sex, n (%)**		
Male	74	(61.2)
Female	47	(38.8)
**Series, n (%)**		
Series 1	79	(65.3)
Series 2	42	(34.7)
**Clinical characteristics**		
Tumour diameter, mm, median (range)	60	(10–180)
**Tumour diameter, two tier**		
≤ 70 mm, n/N (%)	77	(63.6)
> 70 mm, n/N (%)	36	(29.8)
Unknown	8	(6.6)
**Tumour laterality**		
Right	60	(49.6)
Left	59	(48.8)
Unknown	2	(1.7)
**Fuhrman tumour grade, n (%)**		
1	16	(13.2)
2	50	(41.3)
3	34	(28.1)
4	21	(17.4)
**Pathologic T stage, n (%)**^**a**^		
T1/T2	76	(62.8)
T3	41	(33.9)
T4	1	(0.8)
Unknown	3	(2.4)
**Pathologic node stage, n (%)**		
N0	85	(70.2)
N1/N2	8	(6.6)
NX	26	(21.5)
Unknown	2	(1.7)
**Metastatic stage, n (%)**		
M0	80	(66.1)
M1	15	(12.4)
MX	24	(19.8)
Unknown	2	(1.7)
**UICC pathologic stage, n (%)**		
I	7	(5.8)
II	62	(51.2)
III	34	(28.1)
IV	16	(13.2)
NA	2	(1.7)
**DNA sample characteristics**		
Concentration, ng/µl (SD)	145.0	(118.7)
**Maximum fragment length, bp**^**b**^		
200, n (%)	23	(19.0)
300, n (%)	38	(31.4)
400, n (%)	53	(43.8)
500, n (%)	7	(5.8)
Duration of storage, years, mean (SD)	9.8	(3.3)
**Sequencing characteristics**		
***VHL***		
Read depth, x, mean (SD)^c^	57.5	(26.7)
Mutated, n (%)	45	(37.2)
***PBRM1***		
Read depth, x, mean (SD)^c^	42.1	(22.0)
Mutated, n (%)	27	(22.3)
***SETD2***		
Read depth, x, mean (SD)^c^	44.3	(22.6)
Mutated, n (%)	14	(11.6)
***KDM5C***		
Read depth, x, mean (SD)^c^	60.4	(32.4)
Mutated, n (%)	12	(9.9)
***BAP1***		
Read depth, x, mean (SD)^c^	64.4	(34.5)
Mutated, n (%)	7	(5.8)
***TP53***		
Read depth, x, mean (SD)^c^	65.1	(33.2)
Mutated, n (%)	3	(2.5)
***MTOR***		
Read depth, x, mean (SD)^c^	68.2	(35.1)
Mutated, n (%)	3	(2.5)

^a^According to the TNM Classification of Malignant Tumours. Third Edition, Revised Edition^24^.

^b^Longest DNA fragment detected using a DNA fragment ladder test.

^c^Uniquely aligned reads based on the UMI-based custom panel kit (Ovation™ Custom Target Enrichment System, NuGEN, San Carlos, CA, USA).

### Mutations and clinical characteristics

Based on sequencing quality, 121 ccRCC cases were available for further analysis. However, due to missing data, the final number of cases varied slightly per clinical characteristic (Table 2). Of the seven selected genes, four (*VHL*, *PBRM1*, *SETD2* and *KDM5C*) were mutated in at least 10 cases and thus included in our logistic regression models of the association with clinical characteristics (Table 2). In neither the univariable models nor the multivariable-adjusted models did we observe a statistically significant association between tumour characteristics and the mutation status of *VHL*, *PBRM1*, *SETD2* and *KDM5C*.

**Table 2:** Logistic regression models on the association between mutated genes and clinical characteristics. The FDR-adjusted *p*-value was calculated using the Benjamini–Hochberg method. The cut-off for a statistically significant *p*-value and FDR-adjusted *p*-value is 0.05.

Interrogated gene	Characteristic		Mutated		Model 1^a^			Model 2^b^			
			No	Yes	OR	(95% CI)	*p*-value	OR	(95% CI)	*p*-value	FDR adj *p*-value
VHL	Grade	1 + 2	41	25	1	Ref		1	Ref		
		3 + 4	35	20	0.94	(0.45–1.97)	0.864	0.73	(0.31–1.73)	0.480	0.919
	Stage	1 + 2	48	21	1	Ref		1	Ref		
		3 + 4	27	23	1.95	(0.91–4.15)	0.084	1.70	(0.69–4.18)	0.250	0.693
	Tumour size	≤ 70	53	24	1	Ref		1	Ref		
		> 70	19	17	1.98	(0.88–4.45)	0.101	1.96	(0.79–4.86)	0.145	0.693
		Continuous per 10 mm	72	41	1.06	(0.94–1.20)	0.320	1.05	(0.91–1.21)	0.540	0.919
PBRM1	Grade	1 + 2	48	18	1	Ref		1	Ref		
		3 + 4	46	9	0.52	(0.21–1.28)	0.155	0.49	(0.18–1.33)	0.163	0.693
	Stage	1 + 2	52	17	1	Ref		1	Ref		
		3 + 4	40	10	0.76	(0.32–1.85)	0.552	0.94	(0.33–2.70)	0.908	0.966
	Tumour size	≤ 70	59	18	1	Ref		1	Ref		
		> 70	28	8	0.94	(0.36–2.41)	0.892	1.19	(0.42–3.41)	0.747	0.919
		Continuous per 10 mm	87	26	0.96	(0.83–1.11)	0.599	1.00	(0.85–1.19)	0.966	0.966
SETD2	Grade	1 + 2	57	9	1	Ref		1	Ref		
		3 + 4	50	2	0.63	(0.20–2.01)	0.439	0.79	(0.21–3.05)	0.733	0.919
	Stage	1 + 2	61	8	1	Ref		1	Ref		
		3 + 4	44	6	1.04	(0.34–3.21)	0.946	1.30	(0.30–5.69)	0.723	0.919
	Tumour size	≤ 70	69	8	1	Ref		1	Ref		
		> 70	8	4	1.08	(0.30–3.84)	0.908	1.35	(0.33–5.49)	0.676	0.919
		Continuous per 10 mm	77	12	0.86	(0.69–1.09)	0.209	0.85	(0.65–1.12)	0.260	0.693
KDM5C	Grade	1 + 2	59	7	1	Ref		1	Ref		
		3 + 4	50	5	0.84	(0.25–2.82)	0.781	0.38	(0.08–1.72)	0.208	0.693
	Stage	1 + 2	65	4	1	Ref		1	Ref		
		3 + 4	43	7	2.65	(0.73–9.59)	0.139	3.13	(0.67–14.57)	0.147	0.693
	Tumour size	≤ 70	71	6	1	Ref		1	Ref		
		> 70	32	4	1.48	(0.39–5.61)	0.565	1.30	(0.29–5.76)	0.731	0.919
		Continuous per 10 mm	103	10	1.05	(0.87–1.28)	0.599	1.02	(0.80–1.30)	0.879	0.966

^a^Univariable logistic regression model.

^b^Multivariable logistic regression model mutually adjusted for differentiation grade, TNM stage and tumour size (continuous), where applicable.

To assess the association between clinical characteristics and mutation status, we performed sensitivity analyses using Pearson chi-square tests, where applicable (Supplementary Table S1.2). These sensitivity analyses were performed to confirm findings from logistic regression models and gain insight into the associations for the genes for which there were too few cases with a mutation to assess using regression models. Here again, similar to the logistic regression models, we found no association with tumour grade, stage or size for *VHL, PBRM1, SETD2* and *KDM5C*. Nor was any association found between these clinical characteristics and mutations in *BAP1, MTOR* and *TP53*.

### Survival analysis

**Figure 2:**
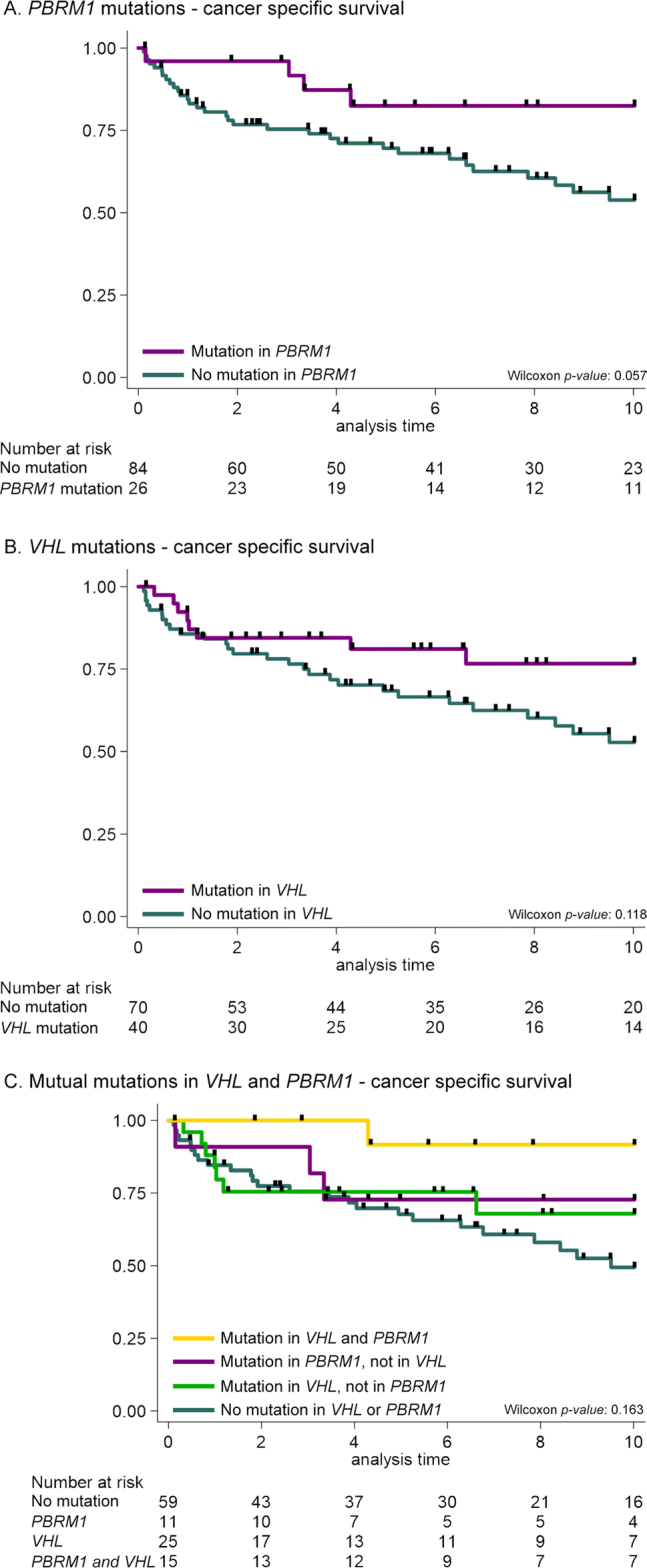
Kaplan–Meier curves for the associations of (**A**) *PBRM1*, (**B**) *VHL* mutations and (**C**) mutual *PBRM1* and *VHL* mutations with ccRCC-specific survival truncated at 10-years of follow-up.

After excluding cases missing clinical information about a priori confounders, we included 110 cases in the survival analyses. Overall, no mutations were identified in 46 cases. Kaplan–Meier curves for the association of mutated *PBRM1* or *VHL* with CSS are displayed in Fig. 2A,B, respectively. Cases with *PBRM1* mutations had a higher CSS than cases without a *PBRM1* mutation, although this association only reached borderline statistical significance (*p*-value = 0.057, Wilcoxon test). Mutations in *VHL* seemed beneficial to CSS, although these results were not statistically significant (*p*-value = 0.118, Wilcoxon test). In age- and sex-adjusted Cox regression models truncated at 10 years of follow-up (model 1, Table 3), we observed an increased CSS for patients with *VHL* mutations [Hazard Ratio (HR) (95% Confidence Interval (CI)] 0.53 (0.24–1.17) or *PBRM1* mutations [HR (95% CI) 0.36 (0.12–1.01)] compared to those without *VHL* or *PBRM1* mutations, although this was again not statistically significant. In the multivariable-adjusted results (model 2, Table 3) that included patient clinical characteristics (tumour grade, size and stage), the association became stronger [HR (95% CI), *VHL* = 0.33 (0.14–0.79), *PBRM1* = 0.25 (0.08–0.79)]. Cases with mutations in *VHL* had a statistically significant increased CSS compared to cases without mutations in *VHL* [HR (95% CI) 0.33 (0.14–0.79)], and we found similar associations for *PBRM1* mutations [HR (95% CI) 0.26 (0.08–0.79)]. After mutual adjustment for other genes, excluding *MTOR* due to the instability of the estimations (model 3, Table 3), the association of *PBRM1* with CSS became slightly stronger [HR (95% CI) 0.17 (0.04–0.66)], while the association of *VHL* remained the same [HR (95% CI) 0.34 (0.13–0.89)]. The statistically significant effect of *VHL* and *PBRM1* mutations on CSS became non-significant after multiple testing correction using the Benjamini–Hochberg method (*q*-values = 0.063 and 0.063, respectively).

**Table 3:** Hazard ratios (HRs) for ccRCC-related deaths truncated at 10 years of follow-up according to genotypes of ccRCC in the Netherlands Cohort Study on diet and cancer. The FDR-adjusted *p*-value was calculated using the Benjamini–Hochberg method. The cut-off for a statistically significant *p*-value and FDR-adjusted *p*-value is 0.05.

Gene mutated		Total no. of cases	No. of ccRCC-related deaths	Survival time, years	Model 1^a^		Model 2^b^		Model 3^c^		*p*-value	FDR adj.* p*-value
					HR	(95% CI)	HR	(95% CI)	HR	(95% CI)		
*VHL*	No	70	28	399	1	Ref	1	Ref	1	Ref		
	Yes	40	8	233	0.53	(0.24–1.17)	0.33	(0.14–0.79)	0.34	(0.13–0.89)	0.028	0.063
*PBRM1*	No	84	32	462	1	Ref	1	Ref	1	Ref		
	Yes	26	4	171	0.36	(0.12–1.01)	0.26	(0.08–0.79)	0.17	(0.04–0.66)	0.010	0.063
*SETD2*	No	98	33	577	1	Ref	1	Ref	1	Ref		
	Yes	12	3	56	1.10	(0.33–3.68)	0.70	(0.16–3.01)	1.04	(0.24–4.45)	0.957	0.957
*KDM5C*	No	100	33	574	1	Ref	1	Ref	1	Ref		
	Yes	10	3	59	1.22	(0.34–4.37)	1.61	(0.44–5.97)	1.43	(0.37–5.43)	0.598	0.672
*BAP1*	No	104	35	593	1	Ref	1	Ref	1	Ref		
	Yes	6	1	40	0.41	(0.06–3.01)	0.36	(0.05–2.74)	0.16	(0.02–1.46)	0.104	0.156
*TP53*	No	107	34	627	1	Ref	1	Ref	1	Ref		
	Yes	3	2	6	5.44	(1.22–24.24)	0.53	(0.09–3.10)	0.43	(0.07–2.86)	0.387	0.498
*MTOR*	No	108	36	622	1	Ref	1	Ref	1	Ref		
	Yes	2	0	11	N/A	–	N/A	–	N/A	–	–	–
**Mutation present in *VHL* and/or *PBRM1***												
No mutation in *VHL* and *PBRM1*		59	25	322	1	Ref	1	Ref	1	Ref		
Mutation in *PBRM1*, not in *VHL*		11	3	67	0.56	(0.17–1.87)	0.28	(0.07–1.11)	0.16	(0.03–0.80)	0.026	0.063
Mutation in *VHL*, not in *PBRM1*		25	7	130	0.73	(0.31–1.70)	0.36	(0.14–0.94)	0.32	(0.11–0.94)	0.039	0.063
Mutation in *VHL* and *PBRM1*		15	1	103	0.15	(0.02–1.09)	0.08	(0.01–0.67)	0.06	(0.01–0.59)	0.015	0.063

^a^Adjusted for age at diagnosis (years) and sex.

^b^Adjusted for age at diagnosis (years), sex, TNM stage, differentiation grade and tumour size.

^c^Adjusted for age at diagnosis (years), sex, TNM stage, differentiation grade and tumour size and mutually adjusted for the other genes. *MTOR* was not included in the model due to the absence of fatal events in participants with an *MTOR* mutation.

The association between other mutated genes and CSS should be interpreted with caution given the low number of ccRCC-related deaths in our study population. Overall, we observed that mutations in most genes were related to an increased survival (with the exceptions of *KDM5C* and *SETD2*)*,* although most of the findings were not statistically significant and were based on a small number of cases (Table 3).

Sensitivity analyses using the complete time of follow-up but truncating the analyses at 5-years follow-up found similar results to those for 10 years of follow-up (Supplementary Tables S1.3, S1.4). Differences were only observed for *SETD2*, with the effect of *SETD2* mutations became less favourable when truncating follow-up time. This effect is likely influenced by the high number of deaths among cases with *SETD2* mutations at the beginning of follow-up, as seen in the Kaplan–Meier curve (Supplementary Fig. S1.1).

### Co-occurrence of VHL and PBRM1 mutations

The inventory of single occurrence or co-occurrence of mutated genes in all 121 cases was dominated by the presence of cases with mutations in *VHL* only, followed by cases with mutations in both *VHL* and *PBRM1*, cases with *PBRM1* mutations only and cases with *VHL* and *SETD2* mutations (Fig. 1). Given the frequent co-occurrence of *VHL* and *PBRM1* mutations, we assessed the influence of having: (1) either no *VHL* or *PBRM1* mutation, (2) a mutated *VHL* gene or *PBRM1* gene, or (3) a mutation in both *VHL* and *PBRM1*. The effect of *VHL* and *PBRM1* mutations is visualized in the Kaplan–Meier curves in Fig. 2C. Here we observed that the highest CSS occurs with presence of mutations in both genes and the lowest CSS with the absence of mutations in both genes. Cox regression analysis assessing the combination of *PBRM1* and *VHL* found similar effect estimates for a *VHL* mutation without a *PBRM1* mutation and for a *PBRM1* mutation without a *VHL* mutation (model 3), as compared to the per-gene analyses [HR (95% CI) 0.32 (0.11–0.94), *p-*value = 0.039 and HR (95% CI) 0.16 (0.03–0.80), *p*-value = 0.026, respectively]. After FDR-adjustment, none of these associations remained statistically significant (Table 3). Even though we observed an even stronger association of the co-occurrence of *VHL* and *PBRM1* mutations with CSS, we cannot draw clear inferences from these results due to limited statistical power. Given the lack of statistical power, we did not assess the less frequent combinations of other mutated genes (Fig. 1B).

## Discussion

We investigated the association between mutations in the seven genes most frequently mutated in ccRCC and CSS in a large-scale prospective cohort featuring long-term follow-up of a population-based selection of cases who underwent surgery for ccRCC but only rarely received adjuvant treatment in the first 3 months post-diagnosis. As expected, *VHL* was the most frequently mutated gene, followed by *PBRM1*, *SETD2*, *KDM5C*, *BAP1*, *MTOR* and *TP53,* with mutation frequencies in line with those reported by the TCGA PanCancer project^6^.

In our study, the presence of mutations in *VHL* and/or *PBRM1* was associated with a favourable CSS, and this association became stronger after adjustment for clinical characteristics. These effects appear to be independent of each other, as similar associations with CSS were observed when assessing patients who had mutations in *VHL* but not *PBRM1,* and vice versa. Prior to FDR-correction, we also observed a statistically significant association of the co-occurrence of *PBRM1* and *VHL* mutations to CSS, but we cannot draw conclusions from these results due to the low number of ccRCC-related deaths in this subgroup. In a previous study^19^, data from the Memorial Sloan Kettering Cancer Center cohort showed no clear effect on CSS for combined *VHL* and *PBRM1* mutations, whereas data from the TCGA suggested a slightly worse CSS.

Supplementary Table S1.1 briefly summarizes CSS data from other studies. In one study, *PBRM1* mutations did show a trend toward favourable CSS, although the association was not statistically significant [HR (95% CI) 0.87 (0.57‒1.34), *p*-value = 0.528]^18^. Only one out of several studies reported that the group of *VHL* mutation carriers showed a trend toward favourable CSS^21^, although this association was also not statistically significant [HR (95% CI) 0.53 (0.25‒1.09), *p*-value = 0.079]. In a study based on ccRCC cases from the NLCS from 1986 to 1997^25^, all diagnosed using a combination of single-stranded conformational polymorphism and direct sequencing, no association between *VHL* mutations and CSS was reported^22^. However, when we restricted our analysis to those cases diagnosed before 1997 to assess differences between the two studies within the same cohort (data not shown), we found results similar to our main analyses. It is noteworthy that, in analyses by Smits et al., the proportion of cases with a mutation in *VHL* is higher (52.5%) than in our study (30.1%) when only looking cases diagnosed up to 1997^22^. This discordance in results may be caused by the relatively conservative variant calling thresholds employed in our study.

It is still unclear how mutations in both *PBRM1* and *VHL* might lead to a more favourable CSS. *PBRM1* and *VHL* mutations are known to be enriched in the early events of ccRCC development, whereas other mutated genes play a role in ccRCC progression^26,27^. This is in line with a previously reported in vivo study that showed that mice deficient for both *VHL* and *PBRM1* develop only low-grade kidney cancer^28^. A statistically significant association has previously been reported between the presence of mutations in *PBRM1* and low-grade ccRCC tumours in patients^29^, but no association was found between mutations in *VHL* and tumour grade^25^. In our study, neither *PBRM1* nor *VHL* was significantly associated with lower grade tumours. In addition, all the survival analyses in our study were adjusted for tumour grade, thus we do not believe that the associations we observed were affected by tumour grade.

While it was not statistically significant, we did observe a potential association between *VHL* mutations and tumour stage and size. There was already some conflicting evidence on these associations, as one prior study observed similar associations^30^, whereas another did not^31^. In our study, multivariable models were adjusted for underlying clinical characteristics, which leads us to believe that the protective effects on CSS we observe for mutations in *VHL* and *PBRM1* are independent of underlying tumour characteristics.

In our study, selective drop-out may have occurred with the exclusion of cases with insufficient read depth. However, chi-square tests detected no difference in the distribution of clinical characteristics between the 252 cases eligible for DNA sequencing and the 121 samples ultimately included in analyses (data not shown). Furthermore, we observed no clear differences in the effect of clinical characteristics on the prognosis of ccRCC in Cox regression models between these two subsets (data not shown). We therefore assume that the effect of selection bias is limited. After drop-out, we had a more-limited sample size and a low prevalence of individual gene mutation frequencies, which led to lower power to detect associations, especially after adjusting for multiple testing. This is apparent in the imprecision of our results, which often show wide CIs. Overall, the frequencies of mutations we observe are in line with the mutation frequencies reported by the TCGA PanCancer project, which adds credibility to our findings^6–8^. We may have been hampered by a limited number of cases with tumour stage IV, as these patients were less likely to receive surgical treatment^22^. Given the years in which tumour classification was performed, our study uses an older tumour grade classification (Fuhrman grading system)^32,33^ and tumour staging system (UICC staging system)^24^. One of the main differences between these classifications was the T-stage threshold between T1 and T2 with regards to tumour size. To remedy this, we combined tumour stages 1 and 2 into one category and adjusted all our models that included tumour stage by tumour size.

This study was subject to limitations. Firstly, no information was available on the methylation status of the genes included in the panel, even though prior studies have implicated promoter methylation in the inactivation of *VHL*. In this study, we only assessed the effect of mutation status, not the effect of hypermethylation on the inactivation of *VHL*. As a result, we may have underestimated the proportion of cases with inactivation of *VHL*. The additional effect on CSS of inactivation through hypermethylation in VHL remains inconclusive based on currently available evidence and thus provides a valuable research opportunity for future studies^22,34,35^. Secondly, due to the limited power in analyses for *SETD2, KDM5C, BAP1, TP53* and *MTOR*, we were unable to assess the influence of specific combination of mutations. This highlights the need for larger-scale studies in order to gain additional insights into mutational patterns.

One of the strengths of our study is that it is based on a large population-based prospective cohort study. Our sample population is homogenously collected from one country, which contrasts with other studies, which have mostly included cohorts from multiple countries with varying healthcare systems or case series from individual specialised centres. In addition, adjuvant treatment may cause difficulties in discerning effects between high and low risk groups in prognostic studies^36^. However, our population received limited adjuvant treatment after surgery. Based on information detailing the treatment plan for the first 3 months post-diagnosis, only one case out of 121 was reported to have received immunotherapy. This study is thus able to provide insights into the effects of somatic mutations on the prognosis, with no or limited influence of preoperative treatment. This may aid researchers and clinicians in gaining a better understanding of the intrinsic mechanism that drives ccRCC prognosis. In recent studies, the incorporation of genomics into risk models for prognostication of RCC has been shown to improve the model prediction. The somatic mutational profile of tumours may enable clinicians to identify patients with an expected poorer prognosis, opening up the opportunity to adjust their treatment strategy accordingly^37,38^.

## Materials and methods

### Study population

Our study population was derived from the NLCS, a prospective cohort study initiated in 1986^23^. As described in detail elsewhere, the NLCS included 120,852 participants aged 55‒69 years at baseline^39^. The entire cohort was followed for cancer incidence by record linkage with the Netherlands Cancer Registry, the Dutch pathology registry (PALGA) and Statistics Netherlands (CBS). The completeness of cancer follow-up through record linkage is estimated to be over 96%^40^.

### Ethics statement

Individuals invited to participate in the NLCS received an invitation letter with details on the study and the use of their data. In addition, they received the baseline questionnaire, which included an envelope for returning toenail clippings. By completing and returning the baseline questionnaire, individuals provided informed consented to participate in the NLCS (response rate 35.5%). Individuals were informed about the possibility to end their participation at any time, at which point all their data would be removed. All methods were performed in accordance with the relevant guidelines and regulations applicable at that time (1986). The institutional review boards of Maastricht University (Maastricht) and the Netherlands Organization for Applied Scientific Research TNO (Zeist) approved the NLCS (on February 2, 1985 and January 6, 1986, respectively). In addition, the institutional review board of Maastricht University (Maastricht) later re-evaluated the original approval of the study protocol and procedures and amended the original approval to include the collection and genetic analysis of tumour blocks (April 12, 2010). Participants did not provide written informed consent to the sharing of data.

### Sample collection

**Figure 3:**
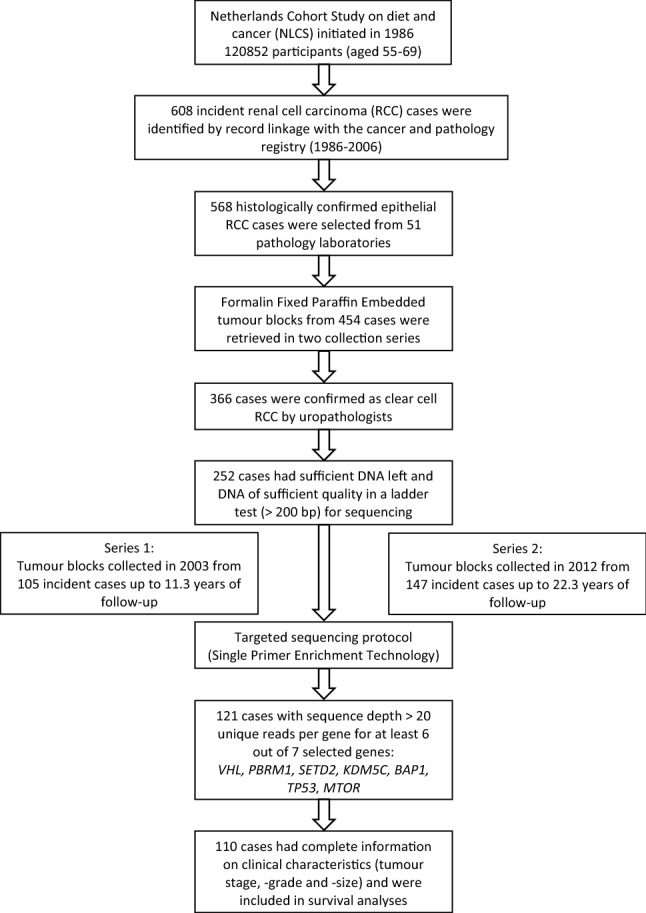
Overview of sample selection of clear cell renal cell carcinoma cases from the Netherlands cohort study on diet and cancer (NCLS), in which DNA collection was performed in two series: Series 1 (collected in 2003) and Series 2 (collected in 2012).

In total, 608 RCC cases were identified in 20.3 years of follow-up from 1986 to 2006 (Fig. 3)^23^. Among these cases, 568 histologically confirmed RCC cases identified through PALGA were selected for the collection of FFPE tissue blocks. FFPE blocks were successfully retrieved from 51 pathology laboratories for 454 cases. Tumour type histology was revised by two experienced uropathologists using the WHO-classification of tumours^3^. Of the 454 cases, 366 were of the ccRCC type^23^. DNA was isolated from collected paraffin tissue blocks in two series. Series 1 included patients diagnosed between 1986 and 1997 and DNA samples collected in 2003^25^. Series 2 included patients diagnosed between 1997 and 2008 and DNA samples collected in 2012^23^. Follow-up was completed up to 31 December 2009. For our study, we selected 252 out of the 366 ccRCC cases based on the availability of sufficient DNA to perform targeted sequencing. In Series 1 samples, the tumour cell fraction was estimated by visual inspection of H&E-stained tissue sections by an uropathologist and varied between 20 and 100% (median 95%). In Series 2, all tumour blocks were subjected to macrodissection of the tumour area in order to enrich for tumour cells prior to DNA analysis. The investigations involving human samples were conducted according to the Declaration of Helsinki^41^ and were covered by the METC permit MEC 85-012-8/ah.

### Targeted sequencing

All DNA samples were subjected to a unique molecular identifier‒based targeted sequencing approach based on Single Primer Enrichment Technology (Ovation™ Custom Target Enrichment System, NuGEN, San Carlos, CA, USA). We applied our previously designed custom landing probe panel for enrichment of the consensus coding regions of the 32 genes most frequently mutated in ccRCC, supplemented with 10 genes associated with the VHL/HIF pathway and the (PI3K)-AKT-MTOR pathway in ccRCC^42,43^.

DNA samples from 252 ccRCC cases were subjected to our sequencing protocol. Aliquots of 500 ng DNA were sheared into 500-bp fragments by Adaptive Focused Acoustics™ (Covaris, Woburn, MA, USA) and subjected to targeted sequencing, as described previously^42^. Enriched libraries were sequenced with the Illumina HISEQ 2500™ (Illumina, San Diego, CA, USA) using single-end next generation sequencing with 100-bp reads. The average read depth per gene varied from 13 to 57.

### Sequence data analysis and somatic mutation identification

Sequencing data were processed using a pipeline following the Genome Analysis Toolkit (GATK) best practice recommendations, with HaplotypeCaller from GATK and FreeBayes used as variant caller^44,45^. Called variants were annotated and filtered to identify true somatic mutations, as described previously^42^. For the current study, we focussed on the six genes with a mutation frequency ≥ 5% in the TCGA PanCancer database, *VHL*, *PBRM1*, *SETD2*, *BAP1*, *MTOR* and *KDM5C*^6^, supplemented with *TP53* because of its high mutation frequency (7%) in the COSMIC database^9^. To minimize the chance of false positive results, samples were only included for further analysis if they had an average read depth of at least 20 unique reads for six genes and a read depth of at least 15 for *TP53*. Next, we determined the somatic variant with the highest mutant allele frequency (MAF) in each patient. Variants with a MAF ≥ 50% of the highest MAF seen on a per sample basis and ≥ 4 mutant reads were considered major variants. This method helps to account for variations in tumour content between samples. The absolute mutant read threshold limits the number of false positives in regions with low coverage. Major clone variants present in more than four samples were excluded because they are likely due to sequencing errors or common population-specific variants. The Integrative Genomic Viewer was used to confirm the authenticity of any doubtful somatic mutations^46^.

The sizeable drop-out (52%) when we restricted our study to samples with an average read depth of at least 20 for six out of the seven genes led us to analyse which sample characteristics were associated with the average read depth of samples under study. This issue is discussed in detail in the Supplementary Data S2.

### Clinical characteristic assessment

Clinical characteristics at diagnosis were derived from excerpts of pathology reports provided by PALGA. From this information, tumour size was assessed based on the largest diameter and was categorized into two tier groups: diameter ≤ 70 mm and diameter > 70 mm^17^. The morphological features of ccRCC and the tumour grade were assessed by two pathologists^23^ using the Fuhrman tumour grade system^32,33^. Tumour stage was provided by the Netherlands Cancer Registry and updated with information from the pathology reports according to the UICC TNM staging system^24^. Information on the cause of death, RCC-related (ICD-10: C64) or other, was obtained from Statistics Netherlands (CBS).

### Statistical analysis

Statistical analysis was performed using Stata statistical software: release 15 (StataCorp., 2015, College Station, TX). The association between mutated genes and tumour stage (stage 1 and 2 vs. stage 3 and 4), tumour grade (grade 1 and 2 vs. grade 3 and 4) and tumour size (≤ 70 mm vs. > 70 mm and continuous) was examined in both univariable and mutually adjusted logistic regression models. We only included genes with at least 10 cases with a mutation in logistic regression analyses. In a sensitivity analysis, we performed univariable analyses for all seven selected genes using chi-square tests. If cells contained five or fewer cases, Fisher’s exact test was used.

We tested the association of each mutated gene with CSS. The survival time (in years) was measured from the time of first diagnosis to the time of death. Cox proportional hazards models were used to estimate HRs and 95% CIs. Analyses were performed using both an age- and sex-adjusted model (model 1) and a multivariable-adjusted model (model 2) that included the following a priori selected confounders: age at diagnosis, sex, tumour grade, tumour size and tumour stage. In model 3, the co-occurrence of mutations in the seven genes was added to model 2. A two-sided *p*-value < 0.05 was considered statistically significant. Multiple testing correction using the Benjamini–Hochberg method^47^ was performed for model 3^48^. False discovery-adjusted *p*-values, i.e. *q*-values, were considered statistically significant if *q* < 0.05. Sensitivity analyses were done using the complete time of follow-up and truncating the follow-up time to 5 years. The proportional hazards assumption was tested using the scaled Schoenfeld residuals and log–log curves^49^. Kaplan–Meier curves and Wilcoxon tests were used to evaluate the cause-specific survival of ccRCC cases with and without each mutated gene. For all cases included in the Cox regression analysis, we also made an inventory of the co-occurrence of mutations involving multiple genes in order to select the most frequent combinations to test for an effect on CSS. In addition, we assessed whether correcting for the collection series had any effect on the survival analysis results. As adjusting for the collection series (Series 1 vs. Series 2) did not largely influence results, and the conclusions remained similar (data not shown), we only present the results unadjusted for collection series.

## Conclusions

In summary, we observe an association between mutations in *VHL* and *PBRM1* and a favourable ccRCC-specific survival. This effect persisted after adjustment for clinical characteristics and the presence of co-occurring mutations in frequently mutated genes in ccRCC. However, the association did not maintain statistical significance after correction for multiple testing, which may be attributed to the low number of samples.

## Supplementary Information

Supplementary Information 1.

Supplementary Information 2.

## Data Availability

The datasets generated during and/or analyzed during the current study are not publicly available because the participants did not provide written informed consent to the sharing of data.
