# Supplementary material for: Evaluation of a seven gene mutational profile as a prognostic factor in a population-based study of clear cell renal cell carcinoma
Source: Sci Rep. 2022 Apr 20;12:6478. doi: 10.1038/s41598-022-10455-x (PMC9021193; doi:10.1038/s41598-022-10455-x)
Supplement: Supplementary file 1 — Supplementary Information 1. [file 41598_2022_10455_MOESM1_ESM.docx]

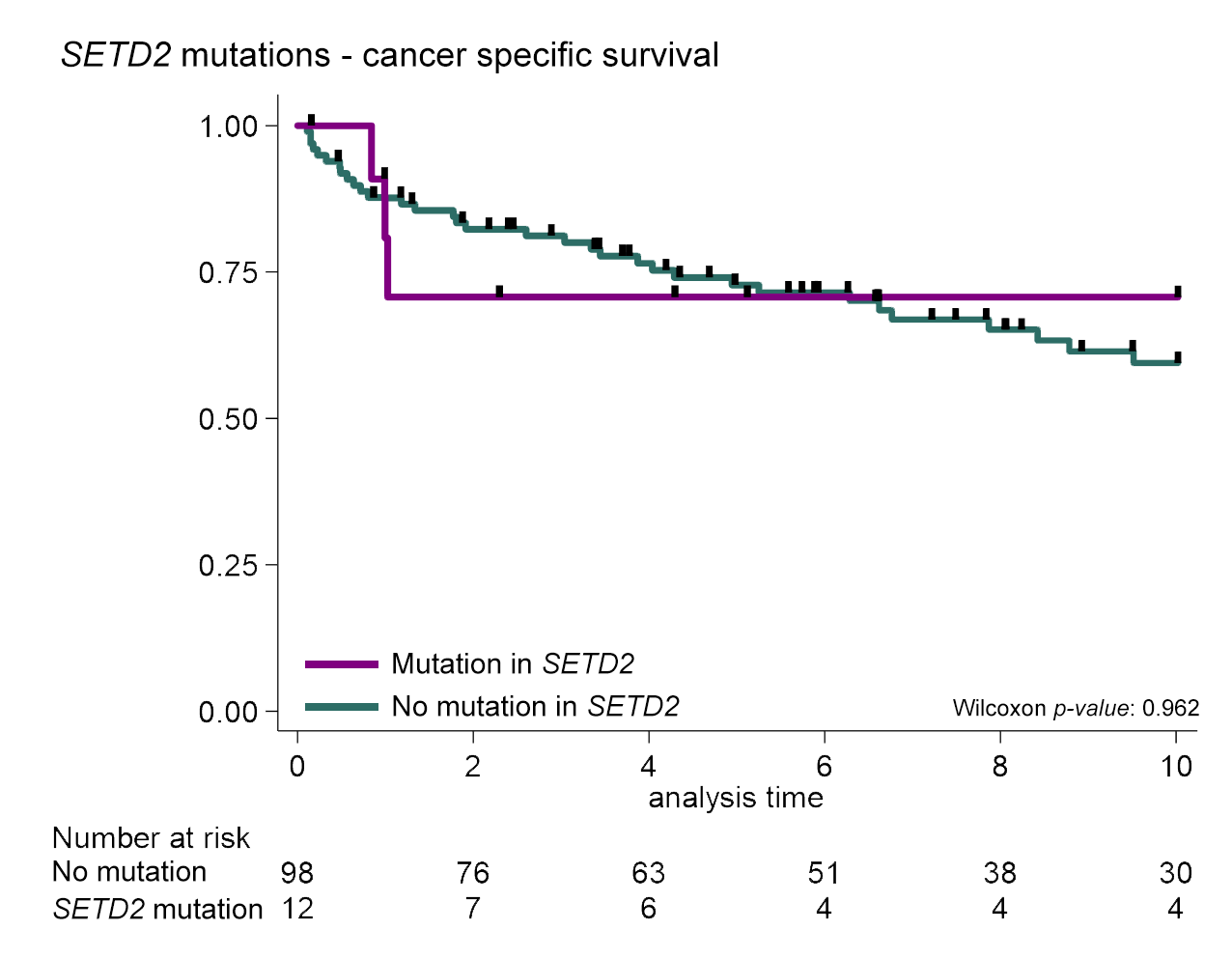


**Supplementary Figure S1.1 -** Kaplan-Meier curve for the association of *SETD2* mutations with ccRCC-specific survival truncated at 10 years of follow-up.

| **Supplementary Table S1.1.** Association between gene-specific mutations and cancer-specific survival of ccRCC patients in previous studies | | | | | | | | | | | |  |
| --- | --- | --- | --- | --- | --- | --- | --- | --- | --- | --- | --- | --- |
| Mutated gene | Study | Sample size | Statistically significant association | HR | 95% CI | Significance | Adjustment variables | Method of analysis | Multiple testing correction | |  |  |
| *BAP1* | Manley *et al*., 2017[1] | 554 | no | 1.29 | 0.85-1.95 | *adj*.*p* = 0.230 | stage, size, grade, and necrosis | multivariable competing risk regression | yes | |  |  |
|  | Hakimi *et al*., 2013a[2] | 119 | yes |  |  | *p* = 0.013 | none | Kaplan-Meier, permutation log rank | no | |  |  |
|  | Hakimi *et al.* 2013b[3] (MSKCC) | 188 | yes | 7.71 | 2.08-28.6 | *P* = 0.002 | none | univariate competing risk regression | no | |  |  |
|  | Hakimi *et al.* 2013b[3] (TCGA) | 421 | yes | 2.21 | 1.35-3.63 | *p* = 0.002 | stage and grade | multivariate competing risk regression | no | |  |  |
|  | Hakimi 2014[4] (TCGA) | 413 | no | 1.27 | 0.76–2.13 | *p* = 0.368  *adj*.*p* = 0.716 | Mayo Clinic stage, size, grade, and necrosis | predictor model, Cox proportional hazards regression | yes | |  |  |
|  |  |  |  |  |  |  |  |  |  | |  |  |
| *KDM5C* | Hakimi 2014[4] (TCGA) | 413 | yes | 1.95 | 1.03–3.7 | *p* = 0.042  *adj*.*p* = 0.656 | Mayo Clinic stage, size, grade, and necrosis | predictor model, Cox proportional hazards regression | yes | |  |  |
|  |  |  |  |  |  |  |  |  |  | |  |  |
| *MTOR* | Hakimi 2014[4] (TCGA) | 413 | no | 1.44 | 0.77–2.71 | *p* = 0.258  *adj*.*p* = 0.656 | Mayo Clinic stage, size, grade, and necrosis | predictor model, Cox proportional hazards regression | yes | |  |  |
|  |  |  |  |  |  |  |  |  |  | |  |  |
| *PBRM1* | Hakimi *et al.* 2013b[3] (MSKCC) | 188 | no |  |  | *p* = 0.93 | none | univariate competing risk regression | no | |  |  |
|  | Hakimi *et al.* 2013b[3] (TCGA) | 421 | no |  |  | *P* = 0.41 | stage and grade | multivariate competing risk regression | no | |  |  |
|  | Hakimi 2014[4] (TCGA) | 413 | no | 0.87 | 0.57–1.34 | *p* = 0.528  *adj*.*p* = 0.853 | Mayo Clinic stage, size, grade, and necrosis | predictor model, Cox proportional hazards regression | yes | |  |  |
|  |  |  |  |  |  |  |  |  |  | |  |  |
| *SETD2* | Hakimi *et al.* 2013b[3] (TCGA) | 421 | yes | 1.68 | 1.04–2.73 | *p* = 0.036 | stage and grade | multivariate competing risk regression | no | |  |  |
|  | Hakimi 2014[4] (TCGA) | 413 | no | 1.02 | 0.6–1.73 | *p* = 0.935  *adj*.*p* = 0.948 | Mayo Clinic stage, size, grade, and necrosis | predictor model, Cox proportional hazards regression | yes | |  |  |
|  |  |  |  |  |  |  |  |  |  | |  |  |
| *TP53* | Manley *et al.* 2017[1] | 554 | yes | 2.23 | 1.27–3.92 | *adj*.*p* = 0.005 | stage, size, grade, and necrosis | multivariable competing risk regression | yes | |  |  |
|  | Hakimi 2014[4] (TCGA) | 413 | no | 2.23 | 0.9–5.52 | *p* = 0.084  *adj*.*p* = 0.656 | Mayo Clinic stage, size, grade, and necrosis | predictor model, Cox proportional hazards regression | yes | |  |  |
|  |  |  |  |  |  |  |  |  | |  | | |
| *VHL* | Hakimi 2014[4] (TCGA) | 413 | no | 1.36 | 0.9–2.05 | *p* = 0.144  *adj*.*p* = 0.656 | Mayo Clinic stage, size, grade, and necrosis | predictor model, Cox proportional hazards regression | yes | |  |  |
|  | Hakimi *et al.* 2013b[3] (MSKCC) | 188 | no |  |  | *p* = 0.78 | none | univariate competing risk regression | no | |  |  |
|  | Hakimi *et. al.* 2013b[3] (TCGA) | 421 | no |  |  | *p* = 0.44 | stage and grade | multivariate competing risk regression | no | |  |  |
|  | Patard *et al*., 2008[5] | 100 | borderline | 0.53 | 0.25–1.09 | *p* = 0.079 | none | 2-year survival univariate Cox regression | no | |  |  |
|  | Smits *et al.* 2008[6] | 185 | no | 1.02 | 0.66–1.58 | *adj*.*p* >0.050 | sex, age at diagnosis, cancer stage, nuclear grade, and tumor size | Cox proportional hazard | yes | |  |  |

Studies were included if they compared the cases with a mutation in a gene to cases without a mutation in a gene, and if the population was not restricted to a specific treatment.

**References**

1. Manley BJ, Zabor EC, Casuscelli J et al. Integration of Recurrent Somatic Mutations with Clinical Outcomes: A Pooled Analysis of 1049 Patients with Clear Cell Renal Cell Carcinoma. *Eur Urol Focus* 2017; 3: 421–427.

2. Hakimi AA, Chen YB, Wren J et al. Clinical and pathologic impact of select chromatin-modulating tumor suppressors in clear cell renal cell carcinoma. *Eur Urol* 2013; 63: 848–854.

3. Hakimi AA, Ostrovnaya I, Reva B et al. Adverse outcomes in clear cell renal cell carcinoma with mutations of 3p21 epigenetic regulators BAP1 and SETD2: a report by MSKCC and the KIRC TCGA research network. *Clin Cancer Res* 2013; 19: 3259–3267.

4. Hakimi AA, Mano R, Ciriello G et al. Impact of recurrent copy number alterations and cancer gene mutations on the predictive accuracy of prognostic models in clear cell renal cell carcinoma. *J Urol* 2014; 192: 24–29.

5. Patard JJ, Fergelot P, Karakiewicz PI et al. Low CAIX expression and absence of VHL gene mutation are associated with tumor aggressiveness and poor survival of clear cell renal cell carcinoma. *Int J Cancer* 2008; 123: 395–400.

6. Smits KM, Schouten LJ, van Dijk BA et al. Genetic and epigenetic alterations in the von hippel-lindau gene: the influence on renal cancer prognosis. *Clin Cancer Res* 2008; 14: 782–787.

**Supplementary Table S1.2.** Association between mutated genes and clinical characteristics. The false discovery rate (FDR)-adjusted *p-value* was calculated using the Benjamini Hochberg method. The cut-off for the statistically significant *p-value* and FDR-adjusted p*-value* is 0.05.

| Characteristic |  | *VHL* | | *PBRM1* | | *SETD2* | | *KDM5C* | | *BAP1* | | *TP53* | | *MTOR* | |
| --- | --- | --- | --- | --- | --- | --- | --- | --- | --- | --- | --- | --- | --- | --- | --- |
|  |  | p*-*value | FDR adj.  p*-*value | p*-*value | FDR adj.  p-value | p-value | FDR adj.  p-value | p-value | FDR adj.  p-value | p-value | FDR adj.  p-value | p-value | FDR adj.  p-value | p-value | FDR adj.  p-value |
| tumour grade | 1/2 vs. 3/4 | 0.864 | 0.864 | 0.190 | 0.570 | 0.436 | 1.000 | 0.781 | 0.781 | 0.243 | 0.452 | 0.091 | 0.296 | 0.091 | 0.273 |
|  |  | Pearson  Chi-square | | Fisher's  exact test | | Pearson  Chi-square | | Pearson  Chi-square | | Fisher's  exact test | | Fisher's  exact test | | Fisher's  exact test | |
|  |  |  | |  | |  | |  | |  | |  | |  | |
| tumour stage | 1/2 vs. 3/4 | 0.083 | 0.147 | 0.551 | 0.827 | 0.946 | 1.000 | 0.199 | 0.597 | 0.452 | 0.452 | 0.572 | 0.826 | 0.572 | 0.858 |
|  |  | Pearson  Chi-square | | Pearson  Chi-square | | Pearson  Chi-square | | Fisher's  exact test | | Fisher's  exact test | | Fisher's  exact test | | Fisher's  exact test | |
|  |  |  | |  | |  | |  | |  | |  | |  | |
| tumour size | ≤70 mm vs.  >70 mm | 0.098 | 0.147 | 0.892 | 0.892 | 1.000 | 1.000 | 0.723 | 0.781 | 0.428 | 0.452 | 0.238 | 0.619 | 1.000 | 1.000 |
|  |  | Pearson  Chi-square | | Pearson  Chi-square | | Fisher's  exact test | | Fisher's  exact test | | Fisher's  exact test | | Fisher's  exact test | | Fisher's  exact test | |

**Supplementary Table S1.3.** Hazard ratios for ccRCC-related deaths according to genotypes of ccRCC for the full follow up time (up to 31 December 2009) in the Netherlands Cohort Study on diet and cancer.

|  |  |  |  |  | Model 1 ^a^ | | |  | Model 2 ^b^ | | |  | Model 3 ^c^ | | |
| --- | --- | --- | --- | --- | --- | --- | --- | --- | --- | --- | --- | --- | --- | --- | --- |
| Mutation | | Total no. of cases | No. of ccRCC related deaths | Survival time, years | HR | (95% CI) | | p-value | HR | (95% CI) | | p-value | HR | (95% CI) | |
| *VHL* | No | 70 | 29 | 501 | 1 | Ref. |  |  | 1 | Ref. |  |  | 1 | Ref. |  |
|  | Yes | 40 | 11 | 290 | 0.71 | (0.35- | 1.43) | 0.333 | 0.47 | (0.22- | 1.00) | 0.051 | 0.54 | (0.23- | 1.23) |
|  |  |  |  |  |  |  |  |  |  |  |  |  |  |  |  |
| *PBRM1* | No | 84 | 35 | 570 | 1 | Ref. |  |  | 1 | Ref. |  |  | 1 | Ref. |  |
|  | Yes | 26 | 5 | 222 | 0.40 | (0.16- | 1.03) | 0.059 | 0.29 | (0.10- | 0.81) | 0.018 | 0.22 | (0.07- | 0.70) |
|  |  |  |  |  |  |  |  |  |  |  |  |  |  |  |  |
| *SETD2* | No | 98 | 37 | 720 | 1 | Ref. |  |  | 1 | Ref. |  |  | 1 | Ref. |  |
|  | Yes | 12 | 3 | 72 | 0.96 | (0.29- | 3.17) | 0.940 | 0.57 | (0.14- | 2.42) | 0.448 | 0.69 | (0.16- | 2.93) |
|  |  |  |  |  |  |  |  |  |  |  |  |  |  |  |  |
| *KDM5C* | No | 100 | 37 | 729 | 1 | Ref. |  |  | 1 | Ref. |  |  | 1 | Ref. |  |
|  | Yes | 10 | 3 | 62 | 1.08 | (0.31- | 3.83) | 0.901 | 1.36 | (0.37- | 4.98) | 0.644 | 1.15 | (0.31- | 4.24) |
|  |  |  |  |  |  |  |  |  |  |  |  |  |  |  |  |
| *BAP1* | No | 104 | 39 | 735 | 1 | Ref. |  |  | 1 | Ref. |  |  | 1 | Ref. |  |
|  | Yes | 6 | 1 | 57 | 0.35 | (0.05- | 2.57) | 0.304 | 0.31 | (0.04- | 2.36) | 0.257 | 0.18 | (0.02- | 1.51) |
|  |  |  |  |  |  |  |  |  |  |  |  |  |  |  |  |
| *MTOR* | No | 108 | 40 | 779 | 1 | Ref. |  |  | 1 | Ref. |  |  | 1 | Ref. |  |
|  | Yes | 2 | 0 | 13 | N/A | - |  |  | N/A | - |  |  | N/A | - |  |
|  |  |  |  |  |  |  |  |  |  |  |  |  |  |  |  |
| *TP53* | No | 107 | 38 | 785 | 1 | Ref. |  |  | 1 | Ref. |  |  | 1 | Ref. |  |
|  | Yes | 3 | 2 | 6 | 5.55 | (1.25- | 24.62) | 0.027 | 0.60 | (0.11- | 3.41) | 0.567 | 0.38 | (0.06- | 2.37) |
| Mutation present in *VHL* and/or *PBRM1* | | | |  |  |  |  |  |  |  |  |  |  |  |  |
| No mutation in *VHL* and *PBRM1* | | 59 | 26 | 410 | 1 | Ref. |  |  | 1 | Ref. |  |  | 1 | Ref. |  |
| Mutation in *PBRM1*, not in *VHL* | | 11 | 3 | 90 | 0.51 | (0.15- | 1.69) | 0.271 | 0.29 | (0.08- | 1.11) | 0.070 | 0.18 | (0.04- | 0.83) |
| Mutation in *VHL*, not in *PBRM1* | | 25 | 9 | 159 | 0.89 | (0.41- | 1.91) | 0.761 | 0.50 | (0.21- | 1.17) | 0.108 | 0.50 | (0.20- | 1.24) |
| Mutation in *VHL* and *PBRM1* | | 15 | 2 | 131 | 0.29 | (0.07- | 1.23) | 0.092 | 0.16 | (0.03- | 0.74) | 0.020 | 0.14 | (0.03- | 0.72) |

^a^Adjusted for age at diagnosis (years) and sex

^b^Adjusted for age at diagnosis (years), sex, TNM stage, differentiation grade and tumor size

^c^Adjusted for age at diagnosis (years), sex, TNM stage, differentiation grade, tumor size and mutually adjusted for the other genes. *MTOR* was not included in the model due to the absence of fatal events in participants with an *MTOR* mutation.

**Supplementary Table S1.4.** Hazard ratios for ccRCC-related deaths truncated at 5-years follow-up according to genotypes of ccRCC in the Netherlands Cohort Study on diet and cancer.

|  |  |  |  |  | Model 1 ^a^ | | |  | Model 2 ^b^ | | |  | Model 3 ^c^ | | |
| --- | --- | --- | --- | --- | --- | --- | --- | --- | --- | --- | --- | --- | --- | --- | --- |
| Mutation | | Total no. of cases | No. of ccRCC related deaths | Survival time, years | HR | (95% CI) | |  | HR | (95% CI) | |  | HR | (95% CI) | |
| *VHL* | No | 70 | 21 | 258 | 1 | Ref. |  |  | 1 | Ref. |  |  | 1 | Ref. |  |
|  | Yes | 40 | 7 | 147 | 0.63 | (0.27- | 1.51) |  | 0.41 | (0.16- | 1.05) |  | 0.36 | (0.12- | 1.09) |
|  |  |  |  |  |  |  |  |  |  |  |  |  |  |  |  |
| *PBRM1* | No | 84 | 24 | 298 | 1 | Ref. |  |  | 1 | Ref. |  |  | 1 | Ref. |  |
|  | Yes | 26 | 4 | 108 | 0.50 | (0.17- | 1.45) |  | 0.34 | (0.11- | 1.11) |  | 0.20 | (0.05- | 0.83) |
|  |  |  |  |  |  |  |  |  |  |  |  |  |  |  |  |
| *SETD2* | No | 98 | 25 | 370 | 1 | Ref. |  |  | 1 | Ref. |  |  | 1 | Ref. |  |
|  | Yes | 12 | 3 | 36 | 1.52 | (0.45- | 5.19) |  | 0.97 | (0.22- | 4.22) |  | 1.19 | (0.27- | 5.28) |
|  |  |  |  |  |  |  |  |  |  |  |  |  |  |  |  |
| *KDM5C* | No | 100 | 26 | 368 | 1 | Ref. |  |  | 1 | Ref. |  |  | 1 | Ref. |  |
|  | Yes | 10 | 2 | 37 | 0.87 | (0.19- | 3.99) |  | 1.31 | (0.27- | 6.22) |  | 1.38 | (0.28- | 6.75) |
|  |  |  |  |  |  |  |  |  |  |  |  |  |  |  |  |
| *BAP1* | No | 104 | 27 | 380 | 1 | Ref. |  |  | 1 | Ref. |  |  | 1 | Ref. |  |
|  | Yes | 6 | 1 | 25 | 0.53 | (0.07- | 3.92) |  | 0.42 | (0.05- | 3.25) |  | 0.17 | (0.02- | 1.70) |
|  |  |  |  |  |  |  |  |  |  |  |  |  |  |  |  |
| *TP53* | No | 107 | 26 | 400 | 1 | Ref. |  |  | 1 | Ref. |  |  | 1 | Ref. |  |
|  | Yes | 3 | 2 | 6 | 5.9 | (1.33- | 26.20) |  | 0.50 | (0.08- | 3.18) |  | 0.46 | (0.07- | 3.18) |
|  |  |  |  |  |  |  |  |  |  |  |  |  |  |  |  |
| *MTOR* | No | 108 | 28 | 400 | 1 | Ref. |  |  | 1 | Ref. |  |  | 1 | Ref. |  |
|  | Yes | 2 | 0 | 6 | N/A | - |  |  | N/A | - |  |  | N/A | - |  |
| Mutation present in *VHL* and/or *PBRM1* | | | | |  |  |  |  |  |  |  |  |  |  |  |
| No mutation in *VHL* and *PBRM1* | | 59 | 18 | 214 | 1 | Ref. |  |  | 1 | Ref. |  |  | 1 | Ref. |  |
| Mutation in *PBRM1*, not in *VHL* | | 11 | 3 | 44 | 0.76 | (0.22- | 2.62) |  | 0.31 | (0.07- | 1.30) |  | 0.15 | (0.03- | 0.88) |
| Mutation in *VHL*, not in *PBRM1* | | 25 | 6 | 84 | 0.84 | (0.33- | 2.13) |  | 0.38 | (0.13- | 1.13) |  | 0.31 | (0.09- | 1.10) |
| Mutation in *VHL* and *PBRM1* | | 15 | 1 | 63 | 0.23 | (0.03- | 1.71) |  | 0.15 | (0.02- | 1.19) |  | 0.11 | (0.01- | 0.99) |

^a^Adjusted for age at diagnosis (years) and sex

^b^Adjusted for age at diagnosis (years), sex, TNM stage, differentiation grade and tumor size

^c^Adjusted for age at diagnosis (years), sex, TNM stage, differentiation grade, tumor size and mutually adjusted for the other genes. *MTOR* was not included in the model due to the absence of fatal events in participants with an *MTOR* mutation.
