# Supplementary material for: Evaluation of a seven gene mutational profile as a prognostic factor in a population-based study of clear cell renal cell carcinoma
Source: Sci Rep. 2022 Apr 20;12:6478. doi: 10.1038/s41598-022-10455-x (PMC9021193; doi:10.1038/s41598-022-10455-x)
Supplement: Supplementary file 2 — Supplementary Information 2. [file 41598_2022_10455_MOESM2_ESM.docx]

**Supplementary Data S2**

*Sample characteristic assessment*

The sizeable dropout (52%) upon restricting the study to samples with an average read depth of at least 20 for 6 out of the 7 included genes drove us to analyse which sample characteristics were associated with the average read depth of samples under study. The sample characteristics investigated were DNA stock concentration after isolation (ng/µl), DNA fragment length (per 100bp increment), duration of FFPE tissue storage (years) and estimated tumour cell fraction (%). DNA concentration was quantified using the PicoGreen® assay. Fragment length was defined by the longest observed DNA fragment on a DNA ladder in 100bp increments from 200 to 600bp. The duration of FFPE block storage was calculated by subtracting the date of cancer diagnosis from the date of DNA isolation. The tumour cell fraction was estimated by visual inspection of H&E-stained tissue sections by an uropathologist. Analyses on sample characteristics were stratified on collection series. The impact of sample characteristics on the sequencing quality was assessed using univariable and mutually-adjusted linear regression models using the sequencing information from the complete panel of 42 genes.

Overall, we observed that sequencing quality decreased with every year of storage as an FFPE block prior to DNA isolation. In addition, samples with a higher DNA concentration after DNA isolation performed significantly better in sequencing. Lastly, DNA fragment length, measured per 100bp, also increased sequencing performance per increment. These effects were especially prominent in Series 1, while Series 2 showed less clear cut results. This is likely attributable to the higher variance in sample characteristics in Series 1, which increased the detectability of differences. However, we could not discern the reason for the sizeable dropout of samples in our study.

**Supplementary Table S2.1** – Sample and sequencing characteristics for all sequenced samples and the subset used for analysis in the main article.

|  | All sequenced samples | | |  | Samples selected for 7-gene analysis | | |
| --- | --- | --- | --- | --- | --- | --- | --- |
|  | Overall | Series 1 | Series 2 |  | Overall | Series 1 | Series 2 |
| No. samples, n (%) | 252 (100) | 105 (41.7) | 147 (58.3) |  | 121 (100) | 79 (65.2) | 42 (34.7) |
| ***Sample characteristics*** |  |  |  |  |  |  |  |
| Concentration, µl/ng (SD) | 100.8 (103.0) | 184.9 (109.3) | 40.7 (32.0) |  | 145.0 (118.7) | 199.4 (111.2) | 42.7 (35.1) |
| Fragment length, bp |  |  |  |  |  |  |  |
| 200, n (%) | 55 (21.8) | 34 (32.4) | 21 (14.3) |  | 23 (19.0) | 23 (29.1) | - |
| 300, n (%) | 69 (27.4) | 43 (41.0) | 26 (17.7) |  | 38 (31.4) | 32 (40.5) | 6 (14.3) |
| 400, n (%) | 95 (37.7) | 24 (22.9) | 71 (48.3) |  | 53 (43.8) | 20 (25.3) | 33 (78.6) |
| 500, n (%) | 33 (13.1) | 4 (3.8) | 29 (19.7) |  | 7 (5.8) | 4 (5.0) | 3 (7.1) |
| Year of cancer diagnosis, year (median, range) | 1997  (1986-2008) | 1993  (1986-1997) | 2001  (1988-2008) |  | 1996  (1986-2008) | 1993  (1986-1997) | 2002.5  (1994-2008) |
| Duration of storage prior to isolation, years (SD) | 10.4 (3.9) | 10.1 (3.0) | 10.6 (4.5) |  | 9.8 (3.3) | 9.9 (3.0) | 9.7 (3.8) |
| Tumour percentage, median (range) | - | 95 (20-100) | - |  | - | 92.5 (20-100) | - |
| ***Sequencing characteristics*** |  |  |  |  |  |  |  |
| Target Coverage >20x, % (SD) | 45.9 (28.8) | 61.2 (24.8) | 34.9 (26.6) |  | 71.6 (12.5) | 72.6 (13.0) | 69.7 (11.4) |
| Mean unique read depth,  x (SD)^a, b^ | 39.1 (34.4) | 60.3 (37.7) | 23.9 (21.4) |  | 66.4 (30.5) | 74.4 (32.2) | 51.3 (19.9) |
| *VHL*, x (SD)^a^ | 33.9 (30.0) | 51.3 (32.0) | 21.5 (21.1) |  | 57.5 (26.7) | 63.0 (27.5) | 47.1 (21.9) |
| *PBRM1*, x (SD)^a^ | 24.2 (23.4) | 37.9 (25.9) | 14.4 (15.2) |  | 42.1 (22.0) | 46.9 (23.3) | 33.3 (15.9) |
| *SETD2*, x (SD)^a^ | 25.4 (24.3) | 39.6 (27.0) | 15.3 (15.7) |  | 44.3 (22.6) | 49.3 (24.0) | 34.8 (16.1) |
| *KDM5C*, x (SD)^a^ | 35.0 (33.8) | 53.0 (38.7) | 22.1 (22.3) |  | 60.4 (32.4) | 66.4 (35.2) | 49.1 (22.9) |
| *BAP1*, x (SD)^a^ | 37.1 (35.8) | 56.0 (40.3) | 23.6 (24.7) |  | 64.4 (34.5) | 69.8 (36.7) | 53.2 (27.3) |
| *TP53*, x (SD)^a^ | 38.2 (35.3) | 57.4 (40.5) | 24.4 (22.9) |  | 65.1 (33.2) | 71.4 (36.6) | 53.3 (21.2) |
| *MTOR*, x (SD)^a^ | 39.3 (37.5) | 61.6 (42.5) | 23.4 (22.7) |  | 68.2 (35.1) | 76.9 (37.7) | 52.0 (22.0) |

^a^ Uniquely aligned reads based on the barcoding-based custom panel kit (Ovation^TM^ Custom Target Enrichment System, NuGEN, San Carlos, CA, USA).

^b^ Uniquely aligned reads for all target regions sequenced prior to selection of genes (42 genes).

**Supplementary Table S2.2 -** Linear regression models for the association between coverage and sample characteristics for all 252 sequenced samples.

|  | All sequenced samples (n=252) | | | |  | Samples selected for 7-gene analysis (n=121) | | | |
| --- | --- | --- | --- | --- | --- | --- | --- | --- | --- |
| Average read depth | Univariable | | Mutually adjusted | |  | Univariable | | Mutually adjusted | |
| (x) | Series 1 (n=105) | Series 2 (n=147) | Series 1 (n=105) | Series 2 (n=147) |  | Series 1 (n=79) | Series 2 (n=42) | Series 1 (n=79) | Series 2 (n=42) |
|  | β (95% CI) | β (95% CI) | β (95% CI) | β (95% CI) |  | β (95% CI) | β (95% CI) | β (95% CI) | β (95% CI) |
| Concentration,  per 10 µl/ng | 1.54  (0.94–2.15) | 0.05  (-1.04–1.15) | 1.29  (0.67–1.91) | -0.19  (-1.35–0.97) |  | 1.19  (0.59–1.79) | -2.04  (-3.73– -0.34) | 0.99  (0.38–1.60) | -2.06  (-3.79– -0.34) |
| Fragment length,  per 100 bp | 12.29  (3.88–20.69) | 2.58  (-1.14–6.31) | 8.86  (0.97–16.74) | 2.45  (-1.54–6.45) |  | 9.52  (1.39–17.66) | -2.07  (-15.83–11.68) | 6.58  (-1.19–14.36) | -2.76  (-15.83–10.31) |
| Duration of storage,  years | -3.66  (-5.98– -1.35) | -0.57  (-1.35–0.22) | -1.87  (-4.15–0.41) | -0.49  (-1.28–0.30) |  | -3.27  (-5.57– -0.96) | -0.75  (-2.42–0.91) | -2.14  (-4.41–0.14) | -0.74  (-2.34–0.85) |
| Tumour content,  per 10% | -0.16  (-4.61–4.29) | - | 1.95  (-2.02–5.92) | - |  | 1.42  (-2.78–5.62) | - | 3.28  (-0.51–7.07) | - |
